# “We Were Not Supposed to Live”: Teaching Medical Rhetoric Through the Lens of Older Adults Living With HIV

**DOI:** 10.1093/geroni/igaf122.1789

**Published:** 2025-12-31

**Authors:** Nels Highberg

**Affiliations:** University of Hartford, Avon, Connecticut, United States

## Abstract

The HIV/AIDS epidemic has evolved from a terminal diagnosis to a chronic condition, resulting in a generation of survivors who were “not supposed to live” now navigating healthcare systems as older adults. This presentation examines the unique challenges faced by older adults living with HIV in medical settings and how these experiences can be effectively incorporated into gerontology curricula through medical rhetoric frameworks. Drawing on classical rhetorical analysis (ethos-pathos-logos), this session demonstrates how faculty can facilitate the critical examination of healthcare policies, institutional communications, and medical discourse affecting older HIV-positive adults. As documented in advocacy resources such as those provided by Lambda Legal, particular attention will be given to discrimination in long-term care facilities and healthcare services. The presentation offers strategies for developing and adapting case studies that remain pedagogically effective even in educational environments where DEI content faces restrictions and explores how AI tools can help create or translate case studies to address regional policy variations while maintaining essential learning objectives. Participants will leave with concrete teaching strategies that promote student understanding of healthcare discrimination against older adults with HIV, rhetorical frameworks for analyzing medical texts that transcend political constraints, and adaptable approaches to case-based teaching in changing educational landscapes.

